# Genome Resequencing of Laboratory Stocks of Burkholderia pseudomallei K96243

**DOI:** 10.1128/MRA.01529-18

**Published:** 2019-02-28

**Authors:** Sariqa Wagley, Andrew E. Scott, Philip M. Ireland, Joann L. Prior, Timothy P. Atkins, Gregory J. Bancroft, David J. Studholme, Richard W. Titball

**Affiliations:** a Biosciences, University of Exeter, Devon, United Kingdom; b Defence Science and Technology Laboratory, Salisbury, Wiltshire, United Kingdom; c London School of Hygiene & Tropical Medicine, London, United Kingdom; Loyola University Chicago

## Abstract

We have resequenced the genomes of four Burkholderia pseudomallei K96243 laboratory cultures and compared them to the reported genome sequence that was published in 2004. Compared with the reference genome, these laboratory cultures harbored up to 42 single-nucleotide variants and up to 11 indels, including a 31.7-kb deletion in one culture.

## ANNOUNCEMENT

Burkholderia pseudomallei causes melioidosis, a bacterial disease of humans and animals (1). It is also a potential biothreat agent (2), and a panel of strains, including K96243, has been proposed to have potential countermeasures to melioidosis (2). Strain K96243 was originally isolated in 1996 from a 34-year-old female diabetic patient in Khon Kaen Hospital in Thailand (3). Since then, this strain has been extensively studied and passed between laboratories around the world. We genome sequenced cultures of strain K96243 with different passage histories held at different laboratories, namely, two from the Defense Science and Technology Laboratory (Dstl) and one each from the University of Exeter (UoE) and the London School of Hygiene and Tropical Medicine (LSHTM). Bacteria were grown with aeration in Luria-Bertani broth at 37°C for 24 h. DNA was extracted using a Genomic-tip 100/G kit (Qiagen Ltd.) following the manufacturer’s instructions. DNA was concentrated using a GeneRead kit (lot no. 145025210), and end repair and adenylation of fragments were carried out using a NEXTflex rapid DNA-seq kit (catalog no. 5144-02) according to the manufacturer’s instructions. Purification and concentration of the PCR-amplified library were carried out according to the GeneRead kit instructions.

The genome sequences shown in Table 1 were determined using 100-bp paired-end libraries with the Illumina HiSeq 2500 system. Quality and adapter trimming were performed using TrimGalore version 0.3.7 (https://www.bioinformatics.babraham.ac.uk/projects/trim_galore/) with the options “—q30 –paired.” TrimGalore uses CutAdapt version 1.15 (4). We used the “mem” algorithm in Burrow-Wheeler aligner (BWA) version 0.7.12-r1039 (5) to align the trimmed reads to the strain K96243 reference genome sequence already available (GenBank accession no. BX571965 and BX571966) (6). The resulting sequence alignment map (SAM) file was converted to binary alignment map (BAM) format using SAMtools version 0.1.19-96b5f2294a (7) with the command line options “view -bS -q 1.” We called variants using Pilon version 1.22 (8) with the options “-Xmx16G –changes –vcf –tracks” and checked the variants using Integrated Genome Viewer version 2.3.78 (9) with its default settings.

**TABLE 1:** Characteristics of resequenced K96243 cultures^*a*^

Strain	SRA accession no.	No. of reads	Coverage (×)	No. of SNVs	No. of indels
K96243-UoE	SRS3855208	5,633,150	41	33	9
K96243-LSTHM	SRS3855207	722,584	18	39	11
K96243-Dstl-1	SRS3855209	905,984	24	42	9
K96243-Dstl-2	SRS3855206	4,534,902	39	41	9

a SNVs and indels are in comparison to the K96243 genome sequences in GenBank (accession no. NC_006350 and NC_006351).

In total, compared with the published sequence, we found 60 single-nucleotide variants (SNVs) across the 4 resequenced cultures (Table 1), and 29 of these SNVs were previously reported (2). At 21 sites, the same SNV was present in all resequenced cultures, suggesting errors in the reference genome. Many of the SNVs were colocated in a 260-bp GC-rich region which may be difficult to sequence or may be hypermutable.

We identified 19 indels ranging from 1 nucleotide (nt) to 33.7 kb across the 4 resequenced cultures. At 5 sites, the same indel was present in all resequenced cultures, suggesting errors in the reference genome. A 31.7-kb region was deleted from chromosome 1 (nucleotide position 1439846 to 1471563; BPSL1247 to BPSL1269) of the UoE culture. This region did not correspond to any of the previously reported genome islands (3) and was not flanked by insertion sequence (IS) elements. It includes 5 hypothetical proteins and a cluster of 5 genes predicted to be involved in cytochrome oxidase-related functions (BPSL1256 to BPSL1257 and BPSL1259 to BPSL1261). It is possible that this region plays a role in electron transport.

Other workers have reported genome plasticity and diversity between different isolates of B. pseudomallei (10), and a recent study reported that, of a number of B. pseudomallei isolates resequenced, strain K96243 showed the greatest divergence from the deposited sequence (2).

The microevolution of B. pseudomallei during infection has previously been reported, with 8 SNVs and 6 small-scale (up to 56 nucleotides [nt]) indels differentiating these variants (11). In addition, derivatives from a single isolate, but with different colony morphologies, showed different virulences (12, 13) and different genetic makeups (14). However, it is reported that genetic differences, including SNVs, do not distinguish these different colony morphotypes (15).

Our findings show that the genetic makeups of laboratory stock cultures of B. pseudomallei strain K96243 are not identical. These findings highlight the need to sequence culture stocks of K96243 held in laboratories before carrying out work with this strain.

### Data availability.

These data have been deposited in DDBJ/ENA/GenBank under BioProject accession no. PRJNA486512. The SRA accession numbers for each strain are SRS3855208 (K96243-Exeter), SRS3855207 (K96243-LSTHM), SRS3855209 (K96243-Dstl-1), and SRS3855206 (K96243-Dstl-2).
